# Supplementary material for: A Comprehensive Assessment of Ultraviolet-Radiation-Induced Mutations in Flammulina filiformis Using Whole-Genome Resequencing
Source: J Fungi (Basel). 2024 Mar 20;10(3):228. doi: 10.3390/jof10030228 (PMC10971301; doi:10.3390/jof10030228)
Supplement: Supplementary file 1 [file jof-10-00228-s001.zip › Supplementary Material S8/KEGG annotation/out/64381550635650.os/KO/out_map/map04742.html]

KEGG PATHWAY: Taste transduction - Reference pathway


|  |  |
| --- | --- |
| **Taste transduction - Reference pathway** |  |

[
Pathway menu
| Organism menu
| Pathway entry
| Show description
| User data mapping
]

Five basic tastes are recognized by humans and most other animals - bitter, sweet, sour, salty and umami. In vertebrates, taste stimuli are detected by taste receptor cells (TRCs). At least three distinct cell types are found in mammalian taste buds : type I cells, type II cells, and type III cells. Type I cells express epithelial sodium channel (ENaC) and are considered to be the major mediator of perception of low salt. In type II cells, transduction of bitter, sweet and umami is mediated by a canonical PLC-beta/IP3-signaling cascade, which culminates in the opening of the TRPM5 ion channel. This produces a depolarization that may allow CALMH1 channels to open and release ATP, which serves as a neurotransmitter to activate closely associated nerve afferents expressing P2X2, P2X3 receptors and adjacent type III cells expressing P2Y4 receptors. Type II taste cells also secrete acetylcholine (ACh) that appears to stimulate muscarinic receptors, specifically M3, on the same or neighboring Type II cells. This muscarinic feedback augments taste-evoked release of ATP. In type III cells, sour taste is initiated when protons enter through apically located proton-selective ion channels: polycystic kidney disease 2-like 1 protein (PKD2L1) and polycystic kidney disease 1-like 3 protein (PKD1L3) channels. Weak acids may also activate sour cells by penetrating the cell membrane and leading to closure of resting K+ channels and membrane depolarization. Further, voltage-gated Ca2+ channels are activated and release vesicular serotonin (5-HT), norepinephrine (NE) and gamma-aminobutyric acid (GABA). 5-HT and GABA provide negative paracrine feedback onto receptor cells by activating 5-HT1A and GABAA, GABAB receptors, respectively. 5-HT also functions as a transmitter between presynaptic cells and the sensory afferent.

|  |  |
| --- | --- |
| Reference pathway | 100% |
